# A Refined Vestibular Romberg Test to Differentiate Somatosensory from Vestibular-Induced Disequilibrium

**DOI:** 10.3390/diagnostics15131621

**Published:** 2025-06-26

**Authors:** Evangelos Anagnostou, Anastasia Gamvroula, Maria Kouvli, Evangelia Karagianni, George Stranjalis, Maria Skoularidou, Theodosis Kalamatianos

**Affiliations:** 1 Department of Neurology, University of Athens, Eginition Hospital, 11528 Athens, Greece; eanagnost@eginitio.uoa.gr (E.A.); anastasiagam@hotmail.com (A.G.); mariadkv94@gmail.com (M.K.); evakara97@gmail.com (E.K.); mskoular@broadinstitute.org (M.S.); 2 Department of Neurosurgery, National and Kapodistrian University of Athens, Evangelismos Hospital, 10676 Athens, Greece; gstranjalis@med.uoa.gr; 3 The Broad Institute of Harvard and M.I.T., Cambridge, MA 02142, USA; 4 Department of Biomedical Engineering, University of West Attica, 12243 Athens, Greece

**Keywords:** Romberg’s test, vestibulopathy, sensory neuropathy, ataxia, posturography

## Abstract

**Background**: The vestibular Romberg test, which assesses the deterioration of balance while standing on rubber foam with closed eyes, is a well-established method in the physical neurological assessment of patients with peripheral vestibulopathy. This study aims to determine whether it can differentiate peripheral vestibulopathy from its main differential diagnosis, namely sensory ataxia, as both conditions typically present with a positive classical Romberg test. **Methods**: Static balance was assessed in three groups: patients with peripheral vestibulopathy, patients with pure sensory neuropathy, and healthy age-matched controls. Participants stood quietly on a force platform under varying visual and proprioceptive feedback conditions. Conventional and advanced postural sway metrics were investigated to establish a quantitative analogy to both the clinical Romberg and vestibular Romberg tests. **Results**: Posturographic analysis revealed that, in contrast to healthy controls, patients with vestibular disorders exhibited higher vestibular Romberg quotient values. However, the classical vestibular Romberg quotient did not show diagnostic discrimination between vestibulopathy and sensory neuropathy patients. This lack of discrimination was mainly due to the increased body sway observed in all patient groups under the “eyes open” condition. Nevertheless, a refined vestibular Romberg quotient—comparing standing on foam versus standing on firm support with eyes closed—was able to reliably distinguish vestibulopathy from sensory ataxia. This distinction was evident in both conventional linear sway and spectral postural sway metrics. **Conclusions**: We conclude that a refined Romberg test, performed solely under conditions of visual deprivation, offers valuable classification potential in differentiating peripheral vestibulopathy not only from healthy controls but also from patients with disequilibrium due to sensory loss.

## 1. Introduction

The cerebellum ensures a bipedal stance by integrating continuous information from proprioception, vision, and the vestibular apparatus. In clinical neurology, the Romberg test is traditionally used to assess postural stability by altering visual input, thereby unmasking disequilibrium in patients with sensory ataxia caused by proprioceptive deficits. The classic Romberg test primarily evaluates proprioceptive function, particularly the integrity of the posterior columns of the spinal cord. It is not a test of cerebellar function, as cerebellar dysfunction typically leads to balance impairment regardless of visual input. Recently, however, the diagnostic reliability of the Romberg test has been debated from both clinical and quantitative posturographic perspectives [1,2].

Patients with unilateral or bilateral peripheral vestibular deficits can maintain an upright stance by compensating with visual and proprioceptive signals. This requires an intact cerebellum capable of employing a central reorganizational strategy to reliably reweight each sensory channel, enhancing the influence of accurate sensory inputs while suppressing conflicting or inaccurate ones. While the Romberg test is standard, it fails to clearly differentiate vestibular from sensory ataxia. This is because both conditions may present with instability when proprioceptive or visual input is removed. Moreover, patients with vestibulopathy can often compensate using vision and proprioception, making it difficult to unmask vestibular deficits using conventional methods. Our study addresses this diagnostic gap by evaluating the “vestibular Romberg test” under conditions that selectively degrade proprioceptive input and vision, allowing for better isolation of vestibular contributions to balance control.

The “vestibular Romberg test” was developed specifically for this patient group, initially designed to highlight their unique deficits [3]. In this test, patients are asked to remain upright with their eyes closed while standing on a specialized foam layer. This setup compromises both vision and proprioception, effectively isolating the (remaining) function of the vestibular system. The vestibular Romberg test has since been utilized in two main ways: (i) as a bedside examination using a simple mattress to assess patients and (ii) through quantitative measurements via static posturography. The latter approach has provided valuable insights into the standing balance of vestibular patients. Posturography is a robust tool for assessing balance impairments in patients with vestibular failure. By quantifying postural sway and evaluating sensory integration, posturography provides insights into the underlying mechanisms of balance dysfunction in these populations. Patients with vestibular failure often experience significant postural instability due to the loss of vestibular input, which is crucial for maintaining balance. Research has shown that individuals with vestibular deficits exhibit increased sway during quiet standing. Early work by Black et al. [3] demonstrated that standing on foam reveals the loss of absolute spatial reference from vestibular input in patients with vestibular dysfunction. More recent studies using larger and more homogenous cohorts confirmed the test’s diagnostic value, reporting good accuracy in distinguishing vestibulopathy from healthy controls [4]. Similarly, another study found that performance on the foam Romberg test was correlated with the incidence of vestibular-related falls [5].

Other studies, however, were less enthusiastic. They found that vestibular patients could only be reliably detected when body sway on foam was evaluated alongside gait assessment [6]. Additionally, other researchers reported that foam posturography failed to distinguish patients with peripheral vestibulopathy from those with cerebellar atrophy [7].

Moreover, posturography offers valuable insights into the balance impairments experienced by patients with sensory neuropathy and sensory ataxia. By quantifying postural control and identifying specific sensory deficits, it aids in diagnosis, guides rehabilitation strategies, and monitors patient progress. Integrating posturographic assessments with clinical evaluations enhances the management of balance disorders in these populations.

In clinical practice, a critical question when examining a patient with gait and trunk ataxia, but without cerebellar signs, is how to differentiate sensory ataxia from vestibular ataxia. The notion that the Romberg and vestibular Romberg tests can distinguish patients with sensory neuropathy (or posterior column dysfunction) from those with vestibulopathy is intuitively appealing but remains entirely untested.

Here, we recruited a cohort of patients with peripheral vestibulopathy and another with pure sensory neuropathy to evaluate the diagnostic value of the vestibular Romberg test. Static posturography was employed, and both classical and advanced analyses of center-of-pressure fluctuations were examined to determine their ability to distinguish sensory ataxia from vestibular ataxia. The expected clinical implication is a more precise and reliable diagnostic tool to guide targeted interventions for patients with balance disorders of unclear etiology.

## 2. Methods

### 2.1. Study and Participants

A prospective study was conducted on three groups of participants:

i. Patients with peripheral unilateral or bilateral vestibulopathy: these individuals were referred to the Outpatient Clinic for Vertigo and Balance Disorders at our hospital.
ii. Patients with pure sensory neuropathy: these individuals were recruited from the electromyography laboratory of the Department of Clinical Neurophysiology.
iii. Age-matched healthy adults: this control group consisted of individuals with no documented history of neurological or vestibular disorders.

All subjects underwent a comprehensive neurological examination. Vestibulopathy patients additionally underwent video head impulse testing (vHIT) and caloric examination of the labyrinths to establish the criteria of uni- or bilateral vestibulopathy [8,9]. Sensory neuropathy patients underwent nerve conduction studies, which confirmed the presence of sensory neuropathy without involvement of motor fibers. This approach ensured a well-defined and comparable cohort for the study. The sensory neuropathy cohort was adopted from our previous study, which investigated the diagnostic performance of the classic Romberg test [2]. Recruitment for the vestibulopathy group began immediately after the conclusion of this earlier project. The study was conducted in accordance with the Declaration of Helsinki and was approved by the Ethics Committee of the Department of Neurology of the University of Athens. An adequate sample size of 30 to 35 participants per group was estimated using a power analysis conducted with the G*Power calculator (Version 3.1.9.7).

### 2.2. Static Posturography

Static balance was assessed by measuring quiet stance for 40 s using a posturographic force platform (Synapsys, Marseille, France). Participants were instructed to stand barefoot with their heels together and their feet at a 45° angle. They were guided to maintain a relaxed posture with their arms hanging by their sides. The platform was equipped with three strain-gauge sensors that continuously tracked the center of foot pressure (COP) excursions. The data were A/D converted at 100 Hz with 12-bit resolution and stored for off-line processing. Subjects were tested in four different conditions:

1. Firm surface, eyes open, fixating on a target 100 cm away (“condition 1”);
2. Firm surface, eyes closed (“condition 2”);
3. Foam pad, eyes open, fixating on a target 100 cm away (“condition 3”);
4. Foam pad, eyes closed (“condition 4”).

A foam pad measuring 13 cm in thickness and with a density of 33 kg/m^3^ was used.

### 2.3. Postural Sway Parameters

The following stabilometric parameters were extracted, either jointly or individually, for both the antero-posterior (AP) and medio-lateral (ML) sway directions:

- Area of the COP sway: this is the surface of the ellipse that contains 90% of the points of the COP excursion.
- AP and ML standard deviation: the standard deviation is the mean deviation with respect to the mean position on the platform.
- AP and ML spectral content: Each 40 s epoch was analyzed by means of a Fast Fourier Transform (FFT), and the resulting spectra were binned into three bands: low frequency (LF) 0–0.5 Hz, middle frequency (MF) 0.5–2 Hz and high frequency (HF) 2–20 Hz). The percentage of energy in each frequency band was taken for further analysis.
- Individual entropy of ML and AP: to obtain discrete ML and AP states, continuous data were min–max normalized and subsequently discretized into an alphabet of ν = 50 bins [10].
- Joint entropy of AP and ML [10].
- The Romberg quotient (RQ): In analogy to the clinical Romberg test, the ratio of the COP sway area, AP, and ML standard deviation of eyes closed to eyes open was calculated while standing on a firm surface (time domain RQ, tRQ). Similarly, the ratio of spectral energy of eyes closed to eyes open defined the frequency domain RQ (fRQ), which was calculated separately for each frequency band. The entropy RQ (eRQ) was also calculated for individual and joint entropies.
- The vestibular Romberg quotient (VRQ) [4,11,12]: While standing on foam, the ratio of the COP sway area, AP, and ML as well as the LF, MF, and HF spectral energy and the individual and joint sway entropies of eyes closed to eyes open was calculated, defining the time domain (tVRQ), the frequency domain (fVRQ), and the entropy (eVRQ) vestibular Romberg quotient.
- The refined vestibular Romberg quotient (rVRQ): This metric was first introduced in the present study to enhance the sensitivity of the foam sway conditions. The rVRQ represents the ratio of the two eyes-closed conditions: eyes closed while standing on foam to eyes closed while standing on firm support.

The above analyses were performed using custom written software in Matlab R2020b (MathWorks, Natick, MA, USA).

### 2.4. Statistical Analyses

As the variables exhibited a deviation from a normal distribution, confirmed by the Kolmogorov–Smirnov normality test, the Kruskal–Wallis test was employed to compare parameters between vestibulopathy patients, sensory neuropathy patients, and controls. Statistical significance was set at a threshold of 0.05. Bonferroni-corrected, post hoc pairwise comparisons were conducted to identify significant differences between groups. The diagnostic performance of the various Romberg quotients was further investigated through Receiver Operating Characteristic (ROC) analysis. Any associations between the reduction in vHIT-gain and significant posturography parameters were tested using Spearman’s correlation analysis. Statistical analyses were conducted using SPSS software version 21.0 (IBM, Armonk, NY, USA). Data visualization was accomplished using SPSS and SRplot [13].

## 3. Results

Thirty-nine patients with unilateral or bilateral peripheral vestibulopathy (mean age: 60.1 ± 13.4 years), thirty patients with pure sensory neuropathy (mean age: 59.7 ± 15.6 years), and thirty age-matched controls (mean age: 55.0 ± 9.7 years) participated in this study (Table 1).

**Figure 1:**
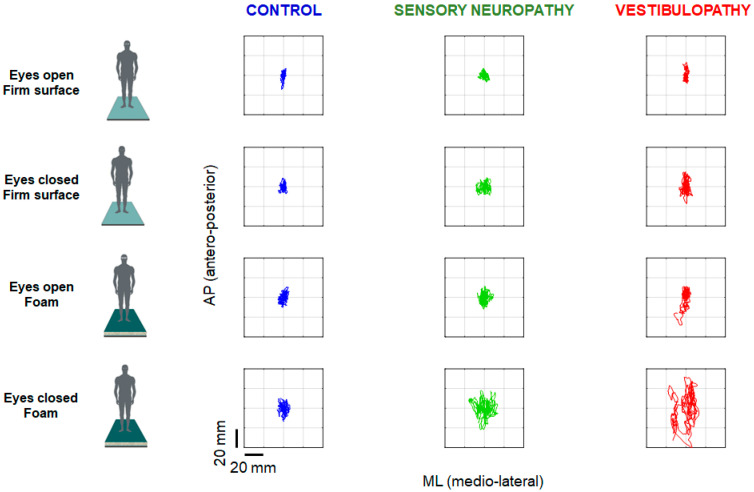
Recordings from a healthy participant (2nd column from **left**, blue) alongside those from a patient with pure sensory neuropathy (3rd column from **left**, green) and a patient with peripheral vestibulopathy (4th column from **left**, red). Comparison of antero-posterior (AP), medio-lateral (ML), and joint (AP vs. ML) postural sway. Each recording spans 40 s and is presented under four distinct sensory feedback conditions.

As anticipated, patients with sensory neuropathy and vestibulopathy exhibited inferior performance compared to controls in most balance tasks across all testing conditions (see example shown in Figure 1). This was evident in both static and dynamic posturographic measurements, underscoring the impact of sensory system impairments on postural control. However, the primary focus of this study was the comparison between eyes-closed and eyes-open balance tasks, as quantified by the Romberg quotient (RQ) and the vestibular Romberg quotient (VRQ), which isolate specific sensory contributions to balance.

As shown in Table 2, differences between patients and controls were less pronounced with respect to RQ and VRQ. Specifically, RQ was abnormal in sensory neuropathy patients in terms of sway area and mediolateral (ML) sway (*p* < 0.05, Kruskal–Wallis test) and showed a trend-level abnormality in antero-posterior (AP) sway (*p* = 0.059, Kruskal–Wallis test).

**Figure 2:**
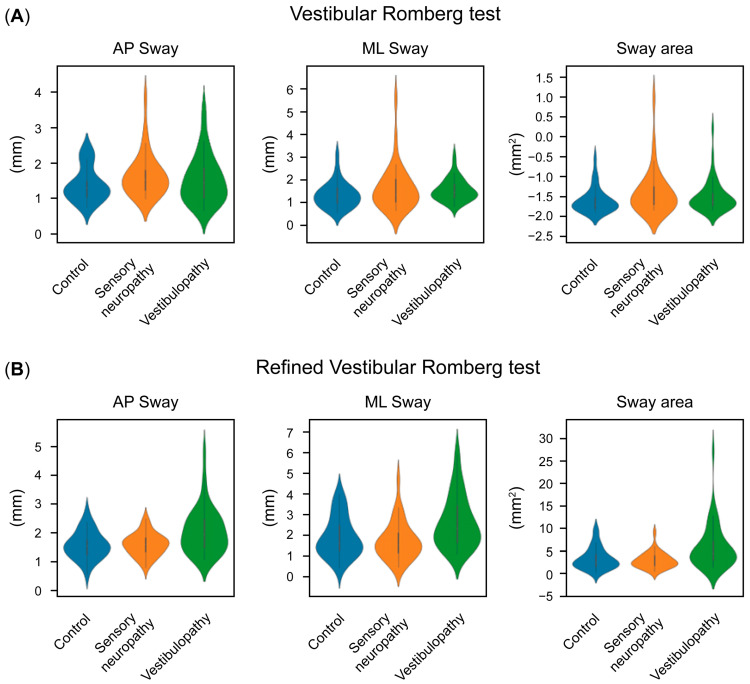
Distribution densities of linear domain vestibular RQs (**A**) and refined vestibular RQs (**B**). Significant differences between sensory neuropathy and vestibulopathy patients are observed only for refined vestibular RQs.

In contrast, VRQ did not reveal significant differences among the groups (Figure 2A). This lack of differentiation is likely due to elevated postural sway in the eyes-open condition, particularly among patient groups, which increased the denominators in all calculated Romberg quotients and consequently masked potential group differences.

Nevertheless, the refined vestibular Romberg quotient (rVRQ) demonstrated clear differences, particularly in vestibulopathy patients (Table 2). Sway area, AP sway, and ML sway showed highly significant differences in the Kruskal–Wallis test (*p* < 0.001, *p* < 0.01, and *p* < 0.001, respectively). Pairwise comparisons further distinguished vestibulopathy patients from both sensory neuropathy patients and controls (Figure 2B).

The rVRQ also revealed significant differences in the frequency domain (Table 3), particularly in low- and middle-frequency antero-posterior (AP) oscillations and high-frequency ML oscillations (Kruskal–Wallis test: *p* < 0.05, *p* < 0.05, and *p* < 0.01, respectively).

More specifically, pairwise group comparisons indicated that vestibulopathy patients exhibited reduced power in the low-frequency band (*p* < 0.05) and increased power in the middle-frequency band (*p* < 0.05) in the AP sway direction compared to sensory neuropathy patients.

Conversely, in the ML direction, sensory neuropathy patients demonstrated enhanced high-frequency oscillations compared to controls (*p* < 0.05), with even greater differences observed relative to vestibulopathy patients (*p* < 0.01). The increased rVRQ values observed in vestibulopathy patients indicate potential utility in clinical settings to improve diagnostic accuracy. The ability to differentiate between vestibulopathy and sensory neuropathy patients using significantly different rVRQ metrics was evaluated through ROC analysis. The areas under the ROC curve (AUC) indicated that the rVRQ of the sway area provided the best classification performance (AUC = 0.751), followed by high-frequency ML sway (AUC = 0.731) and the rVRQ of the standard deviation of ML sway (AUC = 0.726). AUC values for the remaining metrics ranged from 0.6 to 0.7, indicating only fair classification performance and suggesting that no single metric provided perfect separation between groups.

Within the vestibulopathy cohort, correlation analysis showed that a greater reduction in vestibulo-ocular reflex (VOR) gain on the vHIT was associated with a higher rVRQ for sway area (r = −0.430, *p* < 0.01) and AP sway (r = −0.513, *p* < 0.001), supporting the link between reduced vestibular input and postural instability as quantified by rVRQ.

Finally, information-theoretic analyses revealed no significant differences in RQ, VRQ, or rVRQ metrics among vestibulopathy patients, sensory neuropathy patients, and controls. This demonstrates that, despite differences in linear sway magnitude, the complexity of center-of-pressure (COP) fluctuations remained unaffected by the varying sensory feedback conditions (Table 4), suggesting that nonlinear control mechanisms may remain intact despite sensory loss.

## 4. Discussion

Even before the advent of contemporary vestibular testing of high-acceleration VOR using head impulses [14,15,16], posturography had been evaluated against conventional caloric labyrinth testing and was suggested to have equal, if not superior, sensitivity in diagnosing peripheral vestibular failure [17].

However, these findings merely reinforce the well-established fact that vestibular patients are particularly prone to losing their balance, especially under sensory deprivation. This was eloquently illustrated in a letter to the *New England Journal of Medicine* in 1952 by a physician who chose to remain anonymous [18]: “In preparation for shaving, I wrung out the facecloth in steaming hot water, spread it over my hands, and then held it to my face. Thus blindfolded, I suddenly lost my balance and fell sprawling on the floor. Unhurt but surprised, I picked myself up and looked around to see if someone had jostled me. There was no one in the room”.

To be of greater clinical value, however, it would be far more beneficial to employ a test capable of differentiating vestibular dysfunction from other neurological causes of postural disequilibrium.

Posturography is well established procedure for assessing balance impairments in patients with sensory neuropathy and sensory ataxia. By quantifying postural sway and evaluating sensory integration, posturography provides insights into the underlying mechanisms of balance dysfunction in these populations [19,20]. Patients with sensory neuropathy, such as those with diabetic peripheral neuropathy, often experience significant postural instability. This instability is primarily due to the loss of somatosensory input from the lower limbs, which is crucial for maintaining balance. Studies have shown that individuals with diabetic polyneuropathy exhibit increased sway during quiet standing, especially when visual or vestibular cues are diminished, highlighting the reliance on somatosensory feedback for postural control. Further research has demonstrated that the severity of sensory loss correlates with the degree of postural instability. For instance, assessments using force platforms have revealed that patients with greater sensory deficits exhibit larger sway areas and velocities, indicating compromised balance [21]. Posturographic assessments, such as the Sensory Organization Test, have been employed to evaluate balance in individuals with sensory ataxia. These assessments help in identifying the specific sensory deficits contributing to balance impairments [22]. A study involving patients with various forms of ataxia utilized both posturography and clinical balance tests to assess balance control. The findings indicated moderate to strong correlations between posturographic measures and clinical tests like the Berg Balance Scale and the International Cooperative Ataxia Rating Scale. This suggests that combining objective posturographic data with clinical assessments provides a comprehensive understanding of balance dysfunction in sensory ataxia [22]. Rehabilitation programs focusing on balance training have shown promise in improving postural stability in patients with sensory neuropathy and ataxia. For example, a pilot study involving individuals with chronic sensory ataxic neuropathy demonstrated significant improvements in balance and functional outcomes following a 3-week intensive balance and treadmill exercise program [23]. Posturography serves not only as a diagnostic tool but also as a means to monitor progress during rehabilitation. By providing quantitative data on postural sway and sensory integration, clinicians can tailor rehabilitation programs to address specific deficits and track improvements over time [24].

Here, we evaluated the performance of the vestibular Romberg test in distinguishing vestibular unsteadiness from somatosensory unsteadiness. To achieve this, we calculated conventional vestibular Romberg quotients (VRQ) and refined vestibular Romberg quotients (rVRQ) derived from static posturography recordings under four distinct sensory feedback conditions. These metrics were assessed in a cohort of patients with pure sensory neuropathy and another with peripheral vestibulopathy.

Not surprisingly, both patient groups exhibited increased center of pressure (COP) sway compared to controls across most balance tasks and conditions. However, the VRQ failed to reveal significant differences between patients with vestibulopathy and those with sensory neuropathy. The elevated postural sway observed in the eyes-open conditions increased the denominator in all calculated Romberg quotients, leading to the inability to distinguish between the two groups. This limitation was consistent across conventional linear metrics, frequency domain measures, and nonlinear, information-theoretic postural sway metrics.

A more sensitive Romberg quotient was developed by comparing the eyes-closed-on-foam condition to the eyes-closed-on-firm-support condition. This refined VRQ (rVRQ) successfully highlighted differences between vestibular and somatosensory static imbalance. Specifically, the rVRQ revealed marked differences between the two patient groups in sway area, anterior–posterior (AP) sway, and mediolateral (ML) sway. While this finding is straightforward and may have clinical applicability, initial studies on non-apparative bedside assessment of the rVRQ are needed to determine whether the observed increase in postural sway can be reliably detected through clinical observation alone.

Additionally, the rVRQ of the spectral sway content demonstrated distinct characteristics in vestibulopathy patients, including reduced power in the low-frequency band and increased power in the middle-frequency band in the AP direction compared to sensory neuropathy patients. Hence, in all conditions and across both patient and control groups, approximately 80% of the spectral power during quiet standing was observed below 1 Hz—a well-documented finding in previous studies [25,26,27,28]. However, vestibulopathy patients exhibited increased middle-frequency COP fluctuations, possibly indicating an adaptive cerebellar process aimed at enhancing oscillations within the optimal range of the vestibulo-spinal reflex loop [29]. This adaptation may serve to augment stimulation of any remaining intact vestibular afferents.

In contrast, sensory neuropathy patients displayed enhanced high-frequency oscillations in the ML direction compared to those with vestibulopathy. Notably, ML sway reflects a shifting of load between the left and right foot rather than lateral oscillations at the ankles [30]. This may represent a compensatory strategy to enhance plantar somatosensory stimulation in patients with reduced proprioception. A parallel can be drawn to the staggering gait observed in sensory ataxia, where patients lift their legs abnormally high and strike the ground with increased force to compensate for diminished afferent input.

ROC analysis showed strong diagnostic classification performance for significant postural sway metrics, particularly sway area and high-frequency ML sway. Furthermore, patients with more severe vestibular failure, as quantified by vestibulo-ocular reflex (VOR) gain from vHIT recordings, exhibited greater rVRQ values for sway area and AP sway while standing on the force platform.

Advanced analyses of COP fluctuations using information-theoretic methods yielded no significant differences in RQ, VRQ, or rVRQ metrics among vestibulopathy patients, sensory neuropathy patients, and controls. Signal processing involved assessing COP excursions by calculating their Shannon entropy to capture nonlinear features of postural sway, which are often missed by classical metrics [31,32]. Numerous theories concerning complexity and its related concept of entropy have been introduced over time, with ongoing debate among researchers about which measure best captures the maximum amount of information while maintaining interpretability in biological contexts. Traditional Shannon entropy remains one of the most commonly applied methods for assessing the level of uncertainty or disorder within a system, as well as evaluating how efficiently the system conveys information. In the realm of human balance, entropy is thus likely to reflect both the linear and nonlinear patterns in COP oscillations and indicates how close or far these oscillations are from randomness. Generally, higher entropy signifies greater irregularity and complexity, characterizing signals that approach randomness [33,34,35]. Existing studies on postural sway entropy in central nervous system disorders such as Parkinson’s disease, stroke, cerebral palsy, and multiple sclerosis have typically reported decreased entropy in patient groups [36,37,38,39,40]. This suggests a link between centrally induced disequilibrium and a more rigid posture marked by increased regularity in COP fluctuations. However, peripheral disorders, such as the vestibulopathy cohort examined here, have not been investigated using these methods. Our findings suggest that, despite the visibly increased sway magnitude in the eyes-closed-on-foam condition—often apparent even to the naked eye—patients with peripheral vestibular failure maintained postural sway complexity across varying sensory feedback conditions, provided central vestibular processing remained intact. Hence, in a brain where balance and precise spatial navigation are critical functions, central integration of multisensory inputs—namely visual, proprioceptive, semicircular canal, and otolithic signals [41]—appears to operate through a robust computational framework capable of preserving a stable level of functional complexity, even in the presence of partial sensory channel degradation or failure.

This study has several potential limitations. One design-related limitation is that it was conducted at a single center and employed only static posturography, thereby excluding the potential contribution of dynamic posturography in differentiating the disease groups under investigation. This choice, however, was intentional, as we aimed to replicate clinical vestibular Romberg testing, which is inherently static. Moreover, while our findings clearly demonstrate the diagnostic utility of the rVRQ, external validation in an independent cohort is necessary to confirm its clinical applicability. The etiological heterogeneity and the wide range of disease durations within both the sensory neuropathy and vestibulopathy groups may also limit the generalizability of our results. Notably, the substantial variability in disease severity across both patient groups could have influenced between-group comparisons. Finally, we did not formally assess visual acuity, visual field integrity, or muscle strength—all of which may impact static postural control and thus could have influenced the outcomes of the posturographic assessments.

## 5. Conclusions

Our findings support the idea that the classical vestibular Romberg test, while based on the theoretical premise that depriving vision and proprioception should clearly highlight a vestibular deficit, does not sufficiently differentiate vestibular patients from those with sensory ataxia. This may explain the cautious stance of some studies, which suggest that posturography has limited, if any, diagnostic value in cases of peripheral vestibulopathy [42]. However, we have demonstrated that the ratio of sway while standing on foam versus standing on firm support has strong diagnostic potential for peripheral vestibulopathy, provided both assessments are performed with eyes closed. We have termed this test the ‘refined vestibular Romberg test’, which can reliably distinguish not only vestibular patients from healthy controls but also vestibular patients from those suffering from somatosensory disequilibrium due to pure sensory neuropathy. The potential for applying the refined vestibular Romberg test at the bedside, without relying on quantitative sway recordings, offers an exciting avenue for future research.

## Figures and Tables

**Table 1:** Demographics and clinical characteristics of patients.

	Peripheral Vestibulopathy	Sensory Neuropathy
Number	39	30
Age	60.1 ± 13.4 years	59.7 ± 15.6 years
Etiology	Unilateral vestibulopathy *n* = 32 (vestibular neuritis *n* = 26, labyrinthitis *n* = 4, vestibular schwannoma *n* = 2) Bilateral vestibulopathy *n* = 7 (Meniere’s disease *n* = 3, vestibulotoxic drugs *n* = 2, bilateral vestibular schwannoma *n* = 1, unknown *n* = 1)	Diabetes mellitus *n* = 6 Paraneoplastic *n* = 4 Alcohol *n* = 3 Chemotherapy *n* = 2 Sjögren’s syndrome *n* = 2 Unknown *n* = 13

**Table 2:** Romberg and vestibular Romberg quotients for linear time domain sway metrics. AP: antero-posterior; ML: medio-lateral.

		Controls	Sensory Neuropathy	Vestibulopathy
Romberg quotient (median, 25th–75th percentile)	Sway area	1.18 (0.73–1.93)	2.16 (1.02–2.77)	1.38(0.74–1.82)
	AP sway	1.01 (0.76–1.38)	1.32 (1.06–1.62)	1.21 (0.96–1.47)
	ML sway	1.18 (0.87–1.38)	1.53 (1.01–1.94)	1.08 (0.88–1.33)
Vestibular Romberg quotient (median, 25th–75th percentile)	Sway area	1.55 (0.99–2.35)	1.86 (1.51–3.90)	1.78 (1.40–3.21)
	AP sway	1.31 (1.04–1.64)	1.54 (1.23–1.85)	1.45 (0.98–1.89)
	ML sway	1.22 (0.95–1.63)	1.32 (1.02–2.04)	1.43 (1.21–1.78)
Refined vestibular Romberg Quotient (median, 25th–75th percentile)	Sway area	2.32 (1.39–4.17)	2.46 (1.67–4.00)	4.37 (2.89–7.36)
	AP sway	1.51 (1.23–1.78)	1.59 (1.31–1.81)	1.93 (1.42–2.46)
	ML sway	1.61 (1.20–2.53)	1.51 (1.13–2.16)	2.33 (1.56–3.48)

**Table 3:** Romberg and vestibular Romberg quotients for frequency domain sway metrics. AP: antero-posterior; ML: medio-lateral.

		Controls	Sensory Neuropathy	Vestibulopathy
Romberg quotient (median, 25th–75th percentile)	Low frequency AP	0.94 (0.86–1.00)	0.92 (0.78–1.01)	0.96 (0.88–1.03)
	Low frequency ML	0.98 (0.92–1.08)	1.02 (0.88–1.08)	0.98 (0.87–1.02)
	Middle frequency AP	1.91 (1.05–2.51)	1.15 (1.00–2.32)	1.30 (0.91–2.35)
	Middle frequency ML	1.38 (0.85–1.89)	0.86 (0.49–2.07)	1.26 (0.74–2.14)
	High frequency AP	1.05 (0.62–2.19)	1.45 (0.85–2.72)	1.10 (0.70–1.52)
	High frequency ML	1.01 (0.57–1.37)	0.86 (0.52–1.58)	0.98 (0.58–1.36)
Vestibular Romberg quotient (median, 25th–75th percentile)	Low frequency AP	0.91 (0.85–1.00)	0.89 (0.77–1.03)	0.86 (0.75–0.94)
	Low frequency ML	0.98 (0.87–1.03)	0.94 (0.88–1.12)	0.97 (0.91–1.02)
	Middle frequency AP	1.54 (1.05–2.23)	1.64 (0.89–2.56)	1.85 (1.30–3.14)
	Middle frequency ML	1.16 (0.76–2.68)	1.48 (0.62–2.50)	1.43 (0.88–1.87)
	High frequency AP	0.89 (0.67–1.18)	1.43 (0.96–2.03)	1.37 (0.83–1.78)
	High frequency ML	0.72 (0.43–1.3)	1.08 (0.86–2.30)	0.92 (0.59–1.11)
Refined vestibular Romberg quotient (median, 25th–75th percentile)	Low frequency AP	0.93 (0.81–1.05)	1.01 (0.89–1.28)	0.91 (0.77–1.00)
	Low frequency ML	1.04 (1.00–1.14)	1.00 (0.89–1.08)	1.04 (0.96–1.19)
	Middle frequency AP	1.54 (0.84–2.32)	1.00 (0.71–1.48)	1.65 (1.02–2.90)
	Middle frequency ML	1.02 (0.54–1.30)	1.14 (0.64–2.93)	1.04 (0.62–2.41)
	High frequency AP	0.91 (0.60–1.18)	1.10 (0.80–1.98)	0.94 (0.63–1.50)
	High frequency ML	0.44 (0.26–0.76)	0.84 (0.43–2.42)	0.46 (0.26–0.67)

**Table 4:** Romberg and vestibular Romberg quotients for information theoretic sway metrics. AP: antero-posterior; ML: medio-lateral.

		Controls	Sensory Neuropathy	Vestibulopathy
Romberg quotient (median, 25th–75th percentile)	AP entropy	0.99 (0.95–1.05)	1.02 (0.98–1.05)	0.99 (0.97–1.05)
	ML entropy	1.00 (0.97–1.03)	1.00 (0.96–1.04)	1.01 (0.97–1.05)
	Joint entropy	1.00 (0.97–1.03)	1.02 (0.99–1.05)	1.01 (0.98–1.05)
Vestibular Romberg quotient (median, 25th–75th percentile)	AP entropy	1.03 (0.97–1.05)	1.00 (0.97–1.04)	1.00 (0.97–1.05)
	ML entropy	1.00 (0.96–1.05)	0.98 (0.94–1.02)	0.98 (0.95–1.01)
	Joint entropy	1.02 (0.99–1.04)	0.99 (0.98–1.03)	1.00 (0.98–1.03)
Refined vestibular Romberg quotient	AP entropy	1.00 (0.97–1.05)	1.00 (0.98–1.03)	1.00 (0.98–1.03)
	ML entropy	1.01 (0.96–1.05)	0.96 (0.94–1.03)	0.98 (0.94–1.03)
	Joint entropy	1.00 (0.97–1.04)	0.99 (0.96–1.01)	0.99 (0.98–1.02)

## Data Availability

Data available on request due to privacy/ethical restrictions.
